# Different Types of Co-Infection by Contagious Ecthyma Virus, Enteropathogenic *Escherichia coli*, *Mycoplasma conjunctivae*, Ecto- and Endo-Parasites in Four Young Alpine Ibex (*Capra ibex*)

**DOI:** 10.3390/ani14243666

**Published:** 2024-12-19

**Authors:** Lorenzo Domenis, Raffaella Spedicato, Cristina Guidetti, Emanuele Carella, Serena Robetto

**Affiliations:** Istituto Zooprofilattico Sperimentale del Piemonte, Liguria e Valle d’Aosta, Regione Amerique 7G, 11020 Quart, AO, Italy; raffaella.spedicato@izsplv.it (R.S.); emanuele.carella@izsplv.it (E.C.); serena.robetto@izsto.it (S.R.)

**Keywords:** Alpine ibex (*Capra ibex*), contagious ecthyma, ectoparasites, endoparasites *Escherichia coli*, *Mycoplasma conjunctivae*

## Abstract

The research aimed to investigate the perinatal pathology of Alpine ibex (*Capra ibex*) through the study of four young subjects (at the age of 3 to 4 months) found dead in Valle d’Aosta, a region of northwestern Italy. The carcasses were submitted to necropsy followed by an examination of ecto- and endoparasites (ECP and ENP); samples from the gross lesions (in summary, cutaneous papilloma and crusts, ocular discharge, lobular haemorrhagic areas in the lungs, catarrhal–haemorrhagic enterocolitis) were analysed by bacteriological, histopathological, and biomolecular methods to define the etiological agent. The subjects, with various co-infection patterns, were affected by contagious ecthyma virus (ORFV) (agent of a highly diffusive pustular dermatitis transmissible to small ruminants and humans), Enteropathogenic *Escherichia coli* (EPEC) (major etiological agent of infantile diarrhoea especially in developing countries), *Mycoplasma conjunctivae* (MC) (cause of an ocular infection common to goats and sheep), various ECP (ticks and keds) and ENP (lung and intestinal nematodes, and coccidia). This study emphasises the potential role of the Alpine ibex in the transmission of infectious diseases to other animals such as to humans and, secondly, the need to apply diversified analytical approaches, with the commitment of various specialistic skills, in order to define, in detail, the various and frequently overlapping causes that led a free-ranging animal to the death.

## Simple Summary

To deepen the knowledge about the perinatal pathology of the Alpine ibex (*Capra ibex*) and, at the same time, to evaluate its potential role in the interspecific transmission of diseases to other wild and domestic animals, including humans, we present the necropsy findings and subsequent analytic results obtained from four young ibex found dead in Valle d’Aosta, a region of northwestern Italy. The animals, with various co-infection patterns, were affected—showing typical gross and microscopical lesions in target organs and tissues—by contagious ecthyma virus (ORFV) (agent of a highly diffusive pustular dermatitis transmissible to small ruminants and humans), Enteropathogenic *Escherichia coli* (EPEC) (major etiological agent of infantile diarrhoea, especially in developing countries), *Mycoplasma conjunctivae* (MC) (cause of an ocular infection common to goats and sheep), various ectoparasites (ECP) (ticks and keds), and endoparasites (ENPs) (lung and intestinal nematodes, and coccidia).

## 1. Introduction

The Alpine ibex (*Capra ibex*) is a wild mountain ruminant that, although almost completely extinct at the end of the 19th century, has reconquered a large part of its original territories thanks to conservation and awareness programmes, becoming the symbolic animal of the Italian Alps. As for other wild ungulates, the increase in the number has led to an increasing sharing of pastures with domestic ruminants, especially goats and sheep, with a risk of interspecific transmission of several diseases and parasites [1]. For this reason, numerous epidemiological surveillance programmes have monitored the presence of particularly serious infectious diseases in ibex populations, transmissible to humans and animals, such as brucellosis, sarcoptic mange, blue tongue, pestivirus diseases, and paratuberculosis.

In order to further contribute to the knowledge of perinatal pathologies in this alpine ungulate, we describe four cases of co-infection in young ibex variously affected by contagious ecthyma virus (ORFV), Enteropathogenic *Escherichia coli* (EPEC), *Mycoplasma conjunctivae* (MC), ectoparasites (ECPs), and endoparasites (ENPs).

ORFV is an epitheliotropic virus belonging to the *Parapoxviridae* family that causes a highly contagious zoonotic pustular dermatitis, known as contagious ecthyma (CE). The infection has a worldwide distribution, primarily affecting domestic sheep and goats, and it is also reported in many wild ruminants: bighorn sheep (*Ovis canadiensis*), thinhorn sheep (*Ovis dallii*), Alaskan mountain goat (*Oreamnus americanus*), musk ox (*Ovibos moschatos*), reindeer (*Rangifer tarandus*), Sitka black-tailed deer (*Odocoileus hemionus sitkensis*), Sichuan takin (*Budorcas taxicolor tibetana*), chamois (*Rupicapra rupicapra*), Himalayan tahr (*Hemitragus jemlahicus*), serow (*Capricornus crispus*), white-tailed deer (*Odocoileus virginianus*), red deer (*Cervus elaphus*) [2,3,4,5,6,7,8,9,10,11]; in Italian Alpine ibex, CE has been detected several times in the Stelvio National Park, Central Alps [12]. ORFV is a zoonotic agent, and human infection from wild ruminants (especially common in hunters, foresters, veterinarians, biologists, and generally all professional categories most exposed to game) has been reported in America and Europe [10,13,14].

EPEC strains are defined as intimin (codified by the *eae* gene), containing diarrheagenic *Escherichia coli* that do not possess the Shiga toxin genes [15]; they are recognised as pathogens in humans, where they are a major etiological agent of infantile diarrhoea, especially in developing countries [16,17]. EPEC strains have been isolated from the faeces of a variety of domestic and wild animals, including many alpine ruminants such as red deer (*Cervus elaphus*), roe deer (*Capreolus capreolus*), chamois (*Rupicapra rupicapra*), and also Alpine ibex [18,19,20,21,22].

*Mycoplasma conjunctivae* is the causative agent of infectious keratoconjunctivitis (IKC), a highly contagious ocular infection that is common in sheep and goats (where it is also known as the “pinkeye” of domestic small ruminants). In the European Alps, IKC has often been observed in Alpine chamois (*Rupicapra r. rupicapra*) and Alpine ibex (*Capra ibex*), with several past outbreaks. Still, the disease has also been described in other wild Caprinae such as Pyrenean chamois (*Rupicapra r. pyrenaica*), Iberian ibex (*Capra pyrenaica*) and mouflon (*Ovis aries musimon*) in Spain, and *Hemitragus jemlahicus* in New Zealand [23,24,25,26,27].

Finally, ECP and ENP were investigated as a cause of loss of condition and even death in wild fauna; ECP (arthropods as ticks and mites) and ENP (lung and intestinal worms) are often hosted from wild ungulates and are usually detected in different species of ibex [28,29,30,31,32].

The cases reported are particularly interesting considering that it is quite exceptional for a laboratory to receive carcasses of wild Alpine ibex that died at high altitudes, that have a few months of life, and at the same time are in a state of conservation so good as to allow for processing samples and obtaining significant and reliable analytical results, especially for histological and bacteriological outcome.

## 2. Materials and Methods

### 2.1. Study Area and Animal Origin

Four carcasses of young Alpine ibex (*Capra ibex*) were carried to our laboratory for investigation of the cause of death. The animals, all coming from the territory of the Valle d’Aosta Region (north–western Italian Alps), were found dead (or in poor condition and then deceased or euthanized in a short time under veterinary supervision), respectively, in the following period, site, and altitude (Table 1): (1) end of August 2020, Valgrisenche Valley, 1600 m a.s.l.; (2) beginning of August 2021, Valtournenche Valley, 1585 m a.s.l.; (3) at the end of August 2012, Gressoney Valley, 1700 m a.s.l.; (4) at the end of September 2012, Valpelline Valley, 2200 m a.s.l.

### 2.2. Postmortem Examination and Diagnostic Protocol

All carcasses of ibexes were delivered to our laboratory in fresh and good condition. As usual, according to a diagnostic protocol consolidated at our laboratory, biometric surveys were initially conducted to establish the sex, weight, and age of the animals. During necropsy, macroscopic evaluation of the organs was performed, with a detailed description of gross lesions; every damaged organ or tissue (in our case, notably skin, eye, intestine, and lungs) was subjected to parasitological, bacteriological, histological, or molecular–virological analysis depending on the suspected etiological agent.

#### 2.2.1. Parasitological Exams

A general objective examination of the skin surface was performed to verify the presence of ECPs; ECPs were identified using a stereomicroscope (Olympus SZ-40, (Olympus Optical Co., Ltd., Tokyo, Japan) comparing the morphological characteristics with a reference atlas [33,34,35]. For the differential diagnosis of cutaneous proliferative lesions, a skin scratch was routinely taken and then verified using the microscope, after maceration in KOH 20% solution, for the presence of mites (notably *Sarcopets scabiei*), which were always absent in all subjects. ENPs in faeces were examined by flotation, mixing a sample with ZnSO_4_ 100% solution, which after filtration is capable of bringing light eggs (nematodes, cestodes, and coccidia) and heavy eggs (plathelminths) to the surface.

#### 2.2.2. Bacteriological Exams

For skin lesions, Columbia Agar 5% Sheep Blood (CA), Mc Conkey Agar (MC), and RPF Agar were used (at 37 °C for 48 h in aerobiosis), while in the case of enterocolitis, the examination of the main intestinal pathogens was performed. In addition, XLD Agar and BGA Agar at 37 °C for 24 h in aerobiosis were used to detect *Salmonella* spp.; CCDA Agar at 42 °C for 48 h in microaerophilia was used to detect *Campylobacter* spp.; CIN Agar at 30 °C for 24 h in aerobiosis was used to detect *Yersinia* spp. The strains of *Escherichia coli* were confirmed by API 20 E Biomerieux (BioMerieux, Marcy l’Etoile, France) and subsequently typed for the *eae*, *stx1* and *stx2* genes, respectively, encoding the virulence factors intimin, Shiga-toxin 1, and Shiga-toxin 2, by the PCR-RT method described in the ISO/TS 13136/2012 [36] (“Horizontal method for the detection of Shiga toxin-producing *Escherichia coli* (STEC) and the determination of O157, O111, O26, O103 and O145 serogroups”).

#### 2.2.3. Histological Exams

The exam was applied according to the routine method to evaluate the microscopic lesions: fixation of pathological tissue pieces in buffered formalin 4%, paraffin embedding, trimming by microtome at a thickness of 4 µm, haematoxylin–eosin (HE) staining, and final mounting of the slides with Eukitt balsam [37]. The slides were observed in different magnifications with a light microscope Olympus BX60 (Olympus Optical Co., Ltd., Tokyo, Japan), and photos were captured using Olympus Camedia C-4040 Zoom (Olympus Optical Co., Ltd., Tokyo, Japan) and software Olympus DP Soft—Version 3.1.

#### 2.2.4. Molecular Assays

When CE (refer to the results) was suspected, in addition to the routine bacteriological exam, an ORFV-specific PCR-RT was performed on skin lesions as follows. Briefly, DNA was extracted using the ExtractMe genomic DNA kit (Blirt, Gdańsk, Poland) following the manufacturer’s instructions. The detection of ORFV strains was performed by end-point PCR using the Invitrogen™ Platinum™ Quantitative PCR SuperMix-UDG kit (Invitrogen, Waltham, MA, USA) and primers manufactured by Thermo Fisher Scientific (Norristown, PA, USA). The PCR mixture consisted of 500 nM of forward primers (5′-TACACGGAGTTGGCCGTGATCTTGTA-3′), 500 nM of reverse primers (5′-CGCCAAGTACAAGAAGCTGATGA-3′) specifically designed in a previous study [38], 5 μL of 10× buffer, 200 nM of dNTPs, 1.5 μM of MgCl_2_, 0.5 U/μL of Taq polymerase, 3 μL of template, and 34 μL of DNase and RNase-free water in a total volume of 50 μL. The PCR cycling conditions on the Applied Biosystems 2720 Thermal Cycler (Life Technologies) consisted of an initial step at 95 °C for 4 min followed by 40 cycles at 94 °C for 15 s, 64 °C for 30 s, and 72 °C for 30 s [38]. The amplification products were run on a 1.8% agarose gel at 90 V for 60 min and analysed using the Amplisize Molecular Ruler (Bio-Rad, Hercules, CA, USA). PCR amplification of ORFV resulted in a 103 bp product.

In cases with ocular discharge with suspicion of IKC (or even without clinical signs, with the aim of monitoring the infection after past outbreaks as in Case 2), in addition to the routine bacteriological exam, the examination for MC was performed by using PCR as follows. Eye swabs, treated with 0.45 mL of lysis buffer (100 mM Tris–HCl, pH 8.5, 0.05 Tween 20, 0.24 mg/mL proteinase K), were incubated for 60 min at 60 °C and then heated to 95 °C for 15 min to extract DNA. The extracts were tested for the presence of MC DNA with a TaqMan qPCR, according to Vilei et al. [39]. The oligonucleotide primers and TaqMan fluorogenic probe were specific to the conserved 5′-terminal part of gene *lppS* of MC [40]. qPCR reactions were performed by using 2.5 μL of sample, 900 nM of *lppS* forward primer (5′-CAGCTGGTGTAGCACTTTTTGC-3′) and *lppS* reverse primer (5′-TTAACACCTATGCTCTCGTCTTTGA-3′), 300 nM of *lppS* probe (5′-TGCTTCGACTACCAAATATGATGGTGATCCTCT-3′), and TaqMan Universal PCR Master Mix at a 25 μL volume. PCR reactions were run on a StepOne Plus instrument (Thermofisher, Waltham, MA, USA) using the following cycling parameters: 1 step at 50 °C for 2 min and at 95 °C for 10 min, followed by 40 cycles consisting of denaturation at 95 °C for 15 s and extension at 60 °C for 1 min. Real-time fluorescence measurements were taken for each sample by using the StepOne™ Software v2.3 (Thermofisher, Waltham, MA, USA), and the PCR cycle number at which the fluorescent *lppS* signal crossed the threshold was recorded as Ct value (Figure S4).

## 3. Results

All the results are resumed in Table 1.

### 3.1. Biometry

Apart from Subject 2, which was a female, all animals were male. Considering (a) that the Alpine ibex breeding season is approximately between the end of May and the beginning of June, (b) the dates of discovery of the carcasses, (c) both the horn growth of about 4 cm length and the overall morphology (weight, length, height, and teeth), the age was estimated [41] at about 3 months for the subjects numbered 1, 2, and 3 (found towards mid-August), and about 4 months for Subject 4 (found towards the end of September); therefore, all subjects could be classified in the class of age 0, otherwise defined “yearlings” (kids born in the year).

### 3.2. Gross Lesions and Analytical Results

**Figure 1:**
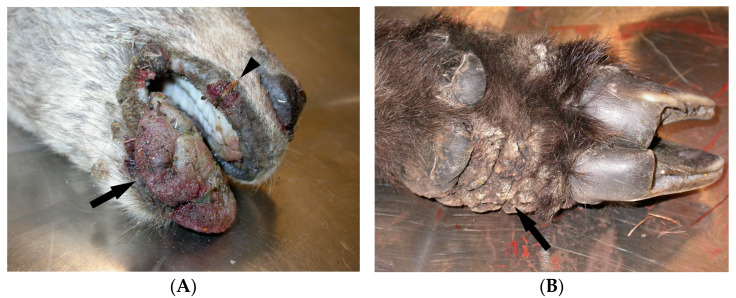

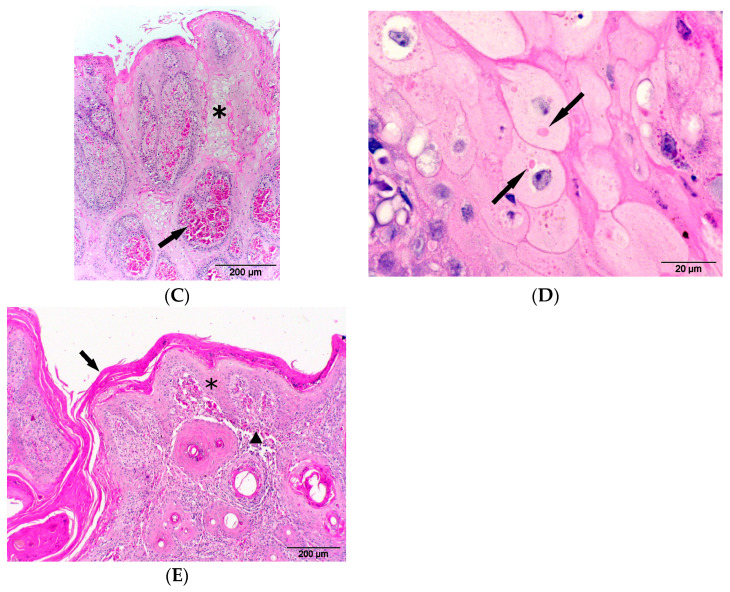
(**A**) Alpine ibex contagious ecthyma. Papillomatous proliferative lesion by ORFV, partially ulcerated and strongly hyperaemic, on the tongue (arrow) and upper lip (arrowhead)—(Subject 1). (**B**) Alpine ibex contagious ecthyma. Typical “cauliflower-like” papillomatous proliferative lesions (arrow) by ORFV, heavily crusted, at the extremities of the limbs, proximal to the nails—(Subject 1). (**C**) Alpine ibex contagious ecthyma. ORFV lesion in the tongue, characterised by epithelial proliferation with spongiosis (asterisk) and severe dilatation of the sub-epithelial vessel network (arrow)—(Subject 1). HE (10×). (**D**) Alpine ibex contagious ecthyma. Acidophilic intra-cytoplasmatic inclusion bodies by ORFV, with clear halo, inside epithelial spongiotic cells of the tongue mucosa (arrows)—(1). HE (100×). (**E**) Alpine ibex contagious ecthyma. Cutaneous proliferative lesions by ORFV, characterised by orthokeratotic hyperkeratosis (arrow), middle acanthosis (asterisk), dilatation of sub-epidermic vessel network, and mixed cells dermal phlogosis (triangle)—(Subject 2). HE (10×).

**Figure 2:**
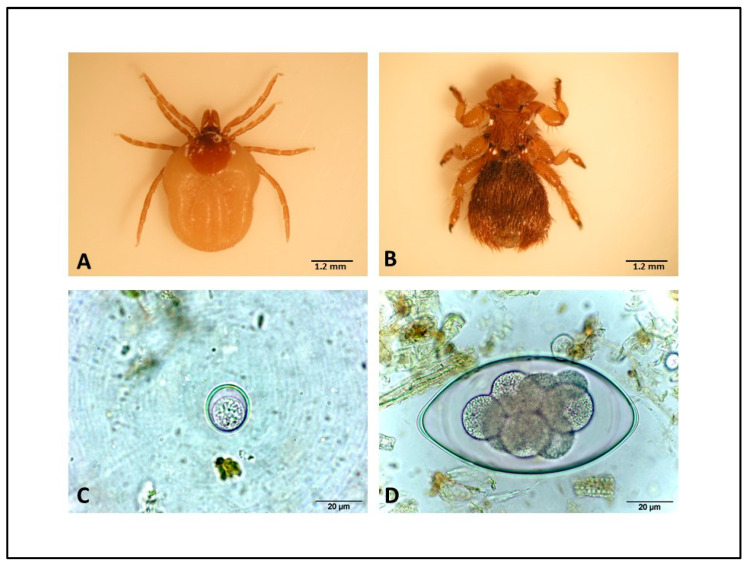
Alpine ibex ecto- and endo-parasites. *Ixodes ricinus* (**A**), *Melophagus rupicaprinus* (**B**), *Eimeria* spp., (**C**), *Nematodirus* spp. (**D**)—(Subjects 1, 2, 4).

Subject number 1: the external physical examination revealed cachexia, the presence of papillomatous proliferative cutaneous lesions (“cauliflower-like”), heavily crusted and sometimes ulcerated, at the level of the tongue (Figure 1A), nostrils, labial rim and limb extremities (Figure 1B), abundant bilateral ocular discharge with focal corneal opacity, and ECPs attributable to *Melophagus rupicaprinus* (Figure 2). After opening the carcass, no macroscopically evident lesions were found on the internal organs. The virological and bacteriological examinations conducted on biopsies of the skin lesions confirmed the suspicion of CE, detecting ORFV (Figure S1) associated with *Staphylococcus aureus*. The histological examination shows parakeratotic and orthokeratotic hyperkeratosis associated with sero-cellular crusts, spongiosis, dyskeratosis, acanthosis, presence of intracytoplasmic acidophilic inclusion bodies surrounded by a clear halo, dermal hyperemia, and mixed sub-epidermal inflammation (Figure 1C–E). Eye swabs tested positive for MC (Figure S4).

Subject number 2: the external physical examination revealed cachexia, the presence of papillomatous proliferative cutaneous lesions, crusted and sometimes ulcerated, at the level of the nostrils, labial rim, and extremities of the limbs, non-severe bilateral ocular discharge, ECPs attributable to mallophages (*Melophagus rupicaprinus*), and ticks (*Ixodes ricinus*) (Figure 2A); the skin lesions were positive for ORFV (Figure S2) without any secondary bacterial infection and the histological examinations showed the same pictures as in Subject 1. After the opening of the carcass, lobular haemorrhagic areas in the lungs and catarrhal haemorrhagic enteritis were observed. Bacteriological and parasitological examinations of the intestine showed the presence of a nonhemolytic EPEC *eae* positive, *stx1*-*stx2* negative strain, associated with ENP, notably coccidia (Figure 2C) and strongyles (with the prevalence of *Nematodirus* spp. and *Ostertagia* spp.) (Figure 2D). The examination for other possible intestinal pathogens (*Salmonella* spp., *Campylobacter* spp., and *Yersinia* spp.), performed for differential diagnosis purposes, was negative. Eye swabs were negative for MC.

Subject number 3: the external physical examination revealed cachexia, the presence of papillomatous proliferative cutaneous lesions, with a crusted surface, at the level of the tongue, neck, sternal region, and extremities of the limbs; the skin lesions were positive for ORFV (Figure S3) without any secondary bacterial infection and with the same microscopical features as Case 1 and 2. After the opening of the carcass, catarrhal enteritis was observed but it was not possible to conduct further analytical investigations. Eye swabs tested positive for MC.

**Figure 3:**
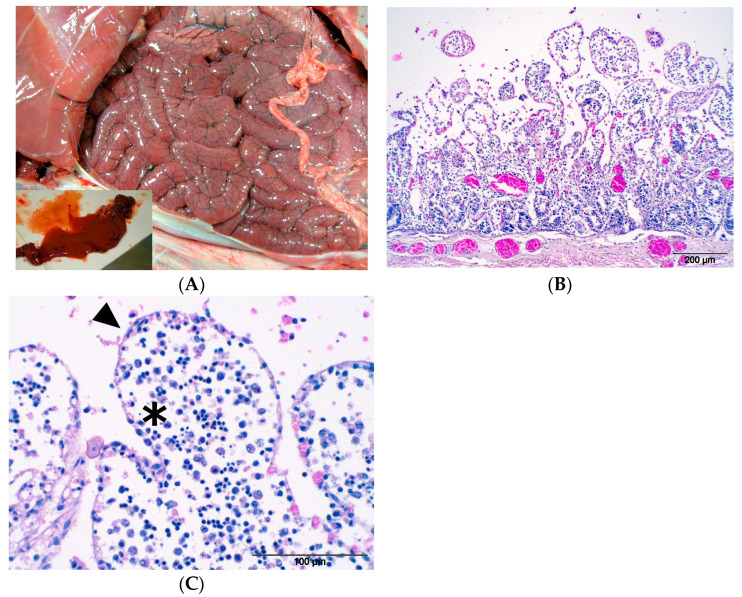
(**A**) Alpine ibex enteritis by EPEC. The intestine appears oedematous, dilated, and congested, with diarrhoeic haemorrhagic content (insert)—(Subject 4). (**B**) Alpine ibex enteritis by EPEC. Acute phlogosis with a severe dilatation of lamina propria and sub-mucosa vessel network—(Subject 4). HE (10×). (**C**) Alpine ibex enteritis by EPEC. Intestinal villi show loss of epithelial surface cells (arrow), oedema, and mixed phlogosis with macrophages, monocytes, and neutrophils (asterisk)—(Subject 4). HE (40×).

**Figure 4:**
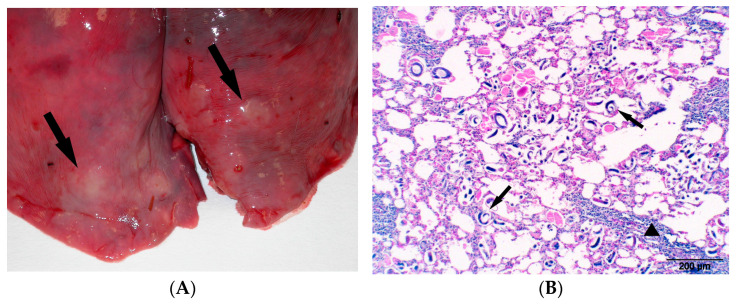
(**A**) Alpine ibex verminous bronchopneumonia. Parasitic lesions inside caudal lung lobes, characterised by multiple-coloured grey foci of consolidated parenchyma (arrows)—(Subject 4). (**B**) Alpine ibex verminous bronchopneumonia. Multiple appreciable nematodes larvae inside alveolar lumina (arrows) and hyperplasia of BALT system (triangle)—(Subject 4). HE (10×).

Subject number 4: the external physical examination revealed moderate cachexia and diarrhoea with faecal contamination of the perianal area. At the opening of the carcass, we observed severe catarrhal haemorrhagic enteritis characterised by dilation and congestion of the small intestine (Figure 3A), with haemorrhagic diarrhoeal stools associated with hyperplasia of the mesenteric lymph nodes and grey consolidated areas in the lung’s parenchyma (Figure 4A). From the intestine, a haemolytic EPEC *eae* positive, *stx1*-*stx2* negative (Figure S5) strain was isolated, associated with the presence of coccidia. In histological examination, intense mixed cell inflammation (lymphocytes, plasma cells, macrophages) and hyperaemia of the intestinal mucosa with de-epithelialization of the apical portion of the villi were observed (Figure 3B,C). The examination for other possible intestinal pathogens (*Salmonella* spp., *Campylobacter* spp., and *Yersinia* spp.), performed for differential diagnosis purposes, was negative. In the lung, typical pictures of verminous bronchopneumonia were detected (larvae and eggs of nematodes, hyperplasia of the smooth muscle septa, lymphohistiocytic inflammation, hyperplasia of BALT system) (Figure 4B). In this case, it was not possible to take ocular swabs for MC.

## 4. Discussion

Among the diseases, CE deserves particular attention as the most prevalent infection, present in 3 out of 4 subjects studied. The CE causative virus belongs to the genus *Parapox* (subfamily *Chordopoxvirinae*, family *Poxviridae*), which comprises four confirmed PPV species [11,12] recognised by the International Committee of Taxonomy of Viruses: the prototype member of virus (ORFV) endemic in most sheep- and goat-raising countries, the bovine papular stomatitis virus (BPSV), the pseudocowpox virus (PCPV) mainly infecting cattle, and Parapoxvirus of Red Deer in New Zealand (PVNZ); this last one is described by Robinson and Mercer [4]. The lesions we have observed in our cases are typical and completely comparable to those described by other authors in domestic and wild ruminants, as in ibex, infected by ORFV. The histological features demonstrate the pathological action of the virus replication in the skin and oral mucosa, which leads to proliferation, vacuolation, and swelling of the epithelial cells, with oedematous and granulomatous inflammation of the derma (cutis) or lamina propria (mucosa), especially when the infection is complicated by overlapping bacterial invasion (*Staphylococcus aureus* in Subject 1 or, as described in other wild ruminants, *Dermatophilus congolensis* or *Trueperella pyogenes* [2,3,4,5,6,7,8,9,10,11,12]). The severe highly vascularization and proliferation observed in the lesions are explained by the expression of proteins homologous to endothelial growth and permeability factor by ORFV, with both being angiogenesis regulators [42]. The painful ulcerated papillomas on the lips and tongue, also observable in domestic lambs or kids, can be particularly severe in young ibex. These animals live in the wild without access to medical treatment or veterinary care and these lesions can significantly impair the ability to suckle and graze, leading to the fatal outcome for starvation, especially in harsh winter months. As mentioned, the ORFV virus is a zoonotic agent that causes a self-limiting disease in humans, usually occurring as a single papule on a finger, hand, or other body part [2]. More important is the risk of spread of ORFV among domestic and wild animals by sharing pastures during the summer season (as frequently happens in our region), since sequencing and comparing the DNA of several isolates has repeatedly demonstrated [3,12] that the virus from many wild ruminants (ibex, chamois, Mountain goat, Dall’s sheep, caribou) clusters with other Orthopoxvirus strains isolated from sheep and goats.

In two out of the four subjects, severe enterocolitis was demonstrated by EPEC strains, positive for the *eae* gene, in association with ENP (coccidia and strongyles); the key feature of EPEC pathogenesis is the production of “attaching and effacing” (A/E) lesions, which are characterised by the intimate adherence of the bacteria to the intestinal epithelium. The *eae* gene, located in the pathogenicity island “locus of enterocyte effacement” (LEE), encodes intimin, an outer membrane protein responsible for the adhesion. Depending on the presence or absence of the EPEC adherence factor (EAF), plasmid EPEC is further divided into two subtypes: typical (tEPEC), more dominant in developing countries, and atypical (aEPEC), more important in developed countries [21]. Despite not having characterised the strains in addition to the *eae* and *stx* genes, the EPEC strains of our cases proved to be particularly aggressive and cytotoxic, causing the complete destruction of the intestinal epithelium with an outcome of haemorrhagic diarrhoea and, besides this, probably lung haemorrhages in Subject 2. EPEC are important human pathogens, representing one of the main causes of infantile diarrhoea in low-income countries to date, but with several outbreaks in the rest of the world mostly linked to food and water consumption [43,44]; considering that *E. coli* can survive for months in the environment, the possibility of transmission to humans by a wide variety of different sources (notably surface water contaminated by wild animal waste) must be taken into account [21,45]. As explained in the introduction, EPEC strains have been reported in many alpine ruminants; therefore, considering that most of these reports generally consist of monitoring based on stool samples without evidence of gastro-intestinal lesions, our cases become particularly interesting as they demonstrate the enteropathogenic action of EPEC in young Alpine ibex, similarly to what commonly happens, with very similar gross and microscopic lesions, for colibacillosis in livestock, especially young calves, lambs, and pigs [46,47].

MC has been detected in two subjects, one with a severe ocular discharge with corneal opacity and the other without evident ocular symptoms. For the last one, it is not possible to establish if it was a healthy carrier or an individual in the incubation phase, or, again, a subject cleared of a previous infection, considering that MC can establish different interactions with its hosts, resulting in very different clinical outcomes, as evident in our cases. When present, clinical signs of IKC are generally associated with ocular damage and inflammation, with visual impairment and blindness until, in more severe stages, staphyloma and perforation of the cornea; these symptoms are often fatal for wild fauna, for which blindness leads to death by starvation or more often by traumatic accident, frequently consequent to falling from a cliff [26]. Small domestic ruminants, mainly sheep, are traditionally assumed as the main reservoir of pathogens, while wild caprine, as chamois, were considered spillover hosts that cannot maintain IKC without the participation of domestic host [1]; however, a separate independent epidemiological cycle has been demonstrated in Iberian ibex [27], and Alpine ibex also seem to be a better candidate in playing a reservoir role complementary to domestic flocks [48]. Because MC is transmitted through direct contact and by flies, the IKC follows a seasonal pattern; like our subjects, both were found dead at the end of August, and the majority of cases were observed in summer, due to the increase in susceptible host densities, consequent to the birth of naive kids, and the abundance of insect vectors [49].

About parasitological findings, ECPs (various arthropods as insects, ticks, and mites) and ENPs as bronchopneumonia nematodes (*Muellerius* spp., *Protostrongylus* spp., *Neostrongylus* spp., *Cystocaulus* spp. and *Dictyocaulus* spp.), coccidia (*Eimeria* spp.), and gastro-intestinal nematodes (notably *Haemonchus* spp., *Marshallagia* spp., *Nematodirus* spp., *Ostertagia* spp., *Trichostrongylus* spp., *Teladorsagia* spp., *Capillaria* spp., *Trichuris* spp.) have been frequently identified in healthy animals among different species of ibex, as in both domestic and other wild ruminants [50,51,52]. Depending on the severity of the infection, age, immunological status, and predisposing factors of the host, progressive negative effects can be caused by different types of parasites, such as respiratory failure and secondary bacterial infections for lung nematodes or acute neonatal diarrhoea for coccidia. Parasites that do not cause disease outbreaks may lead to a constant loss of energetic resources with a decreased growth and survival rate [29]. In our opinion, the presence of ECPs and ENPs, each with their own specific pathogenic action more or less heavy, has certainly contributed to the fatal outcome of the three young Alpine ibexes found to be positive and already stressed by overlapping viral or/and bacterial infections.

## 5. Conclusions

The diagnostic work performed on wild animals found dead is rather complex and difficult; in the absence of any kind of anamnesis, investigation of the causes of death is based exclusively on the anatomical–pathological findings and subsequent laboratory exams. Analyses, especially bacteriological and histological, are often compromised by the poor quality of tissues, especially when the time between death and the finding of the carcass is particularly long, and even more if the weather conditions are adverse (as can happen in summer for high temperatures). The cases we have described, which are particularly lucky regarding the good quality of the young Alpine ibex’ body conservation, confirm what has been gained through our experience; in other words, that wild animals generally die from a fatal combination of different pathological processes (viral and bacteriological infections, parasitic diseases, etc.), often associated with starvation in periods of food scarcity [53]. This reality requires a pathologist to apply diversified analytical approaches, with the commitment of various specialistic skills, in order to define, in detail, the various reasons that led the animal to death. Unlike domestic animals, wildlife is obviously not subject to regular health control and is, therefore, a potential reservoir of zoonoses. In Spain, it has been hypothesised that Iberian ibex could play a role in the maintenance of shared infectious diseases of animal health and conservation at the wildlife–livestock interface [54]. In this perspective, the detection of different pathogens in our study (ORFV, EPEC, MC, ECN, and ENP) reiterates that performing any diagnostic study on Italian Alpine ibex and, in general, wild animal carcasses is extremely important for the welfare of wild populations themselves, both for the monitoring of infections potentially transmissible to humans and finally for the control of the interspecies transmission of pathogens shared between domestic animals and wild fauna.

## Figures and Tables

**Table 1:** Alpine ibex (subjects from 1 to 4). Origin, biometric data, gross lesions sites, and analytical results (M: male; F: female; n.a.: not analysed).

	Subject 1	Subject 2	Subject 3	Subject 4
**Origin**	Valgrisenche Valley	Valtournenche Valley	Gressoney Valley	Valpelline Valley
**Altitude**	1600 m a.s.l.	1585 m a.s.l.	1700 m a.s.l.	2200 m a.s.l.
**Period/Year**	August/2020	August/2021	August/2012	September/2012
**Sex**	M	F	M	M
**Age**	3 m	3 m	3 m	4 m
**ECP**	+	+	−	−
**Lesions site**	Skin, eye, tongue	Skin, intestine	Skin, tongue, intestine	Intestine
**MC**	+	−	+	n. a.
**ORF virus**	+	+	+	−
**EPEC**	/	+	n. a.	+
**ENP**	**−**	**+**	**n. a.**	**+**

## Data Availability

Individual raw data collected are available on request from the corresponding author.

## Supplementary Materials

The following supporting information can be downloaded at https://www.mdpi.com/article/10.3390/ani14243666/s1. Figure S1: Agarose gel electrophoresis of PCR products targeting ORFV for the Subject 1; Figure S2: Agarose gel electrophoresis of PCR products targeting ORFV for the Subject 2; Figure S3: Agarose gel electrophoresis of PCR products targeting ORFV for the Subject 3; Figure S4: PCR amplification plot for *lppS* gene DNA of *Mycoplasma conjunctivae* extracted from ocular swabs of Subject 1; Figure S5: The haemolytic strain of EPEC *eae* positive cultured on Columbia Agar 5% Sheep Blood from the intestine of Subject 4.
