# Supplementary material for: Different Types of Co-Infection by Contagious Ecthyma Virus, Enteropathogenic Escherichia coli, Mycoplasma conjunctivae, Ecto- and Endo-Parasites in Four Young Alpine Ibex (Capra ibex)
Source: Animals (Basel). 2024 Dec 19;14(24):3666. doi: 10.3390/ani14243666 (PMC11727634; doi:10.3390/ani14243666)
Supplement: Supplementary file 1 [file animals-14-03666-s001.zip › animals-3371733-supplementary.pdf]

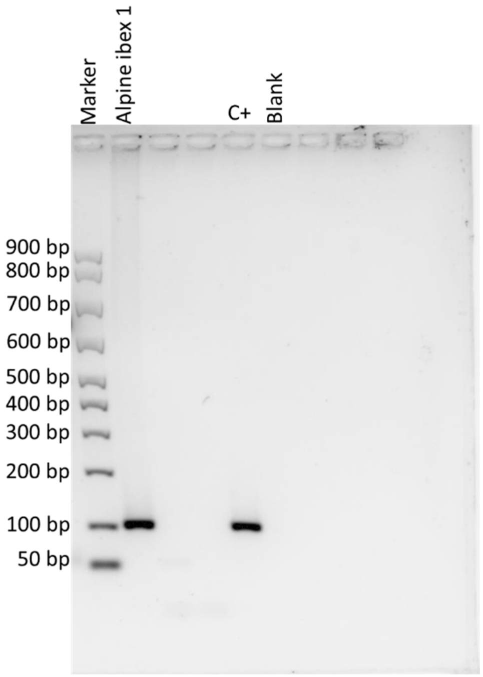

Figure S1: Agarose gel electrophoresis of PCR products targeting ORFV for the Subject 1. Lane 1: DNA marker; Lane 2: Alpine ibex 1; Lane 5: Positive control (C+); Lane 6: Negative control (blank). Lanes 3 and 4 contain unrelated samples that were not considered in this study. PCR amplification of ORFV resulted in a 103 bp product. The gel image has been cropped to highlight the ORFV band. Additional bands corresponding to other pathogens were present but are not shown.

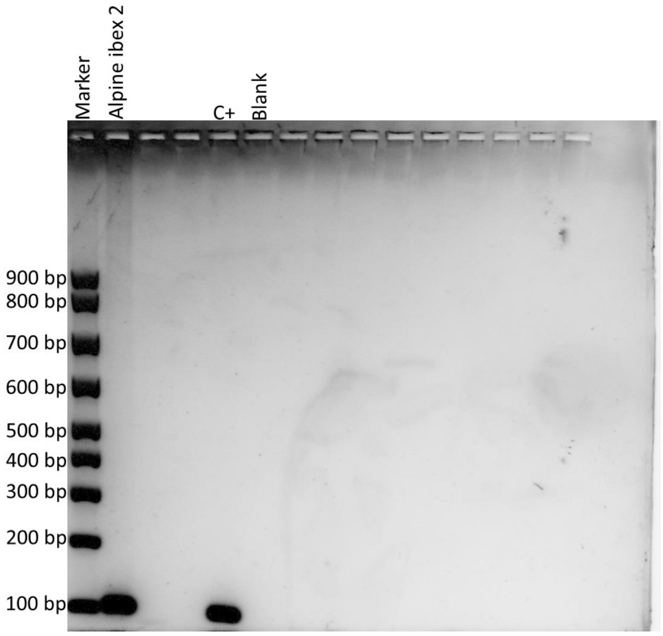

Figure S2: Agarose gel electrophoresis of PCR products targeting ORFV for the Subject 2. Lane 1: DNA marker; Lane 2: Alpine ibex 2; Lane 5: Positive control (C+); Lane 6: Negative control (blank). Lanes 3 and 4 contain unrelated samples not considered in this study. PCR amplification of ORFV resulted in a 103 bp product. The gel image has been cropped to highlight the ORFV band. Additional bands corresponding to other pathogens were present but are not shown.

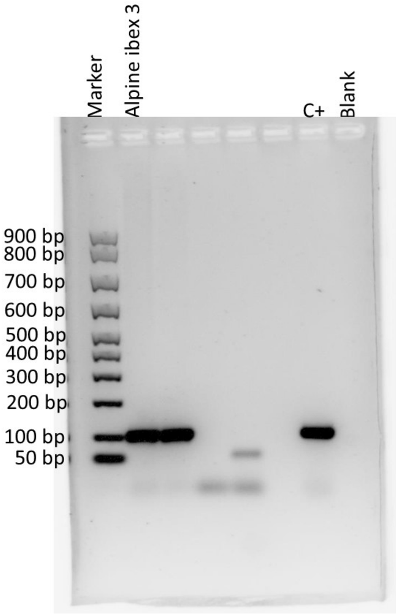

Figure S3: Agarose gel electrophoresis of PCR products targeting ORFV for the Subject 3. Lane 1: DNA marker; Lane 2: Alpine ibex 3; Lane 7: Positive control (C+); Lane 8: Negative control (blank). Lanes 3, 4, 5 and 6 contain unrelated samples not considered in this study. PCR amplification of ORFV resulted in a 103 bp product.

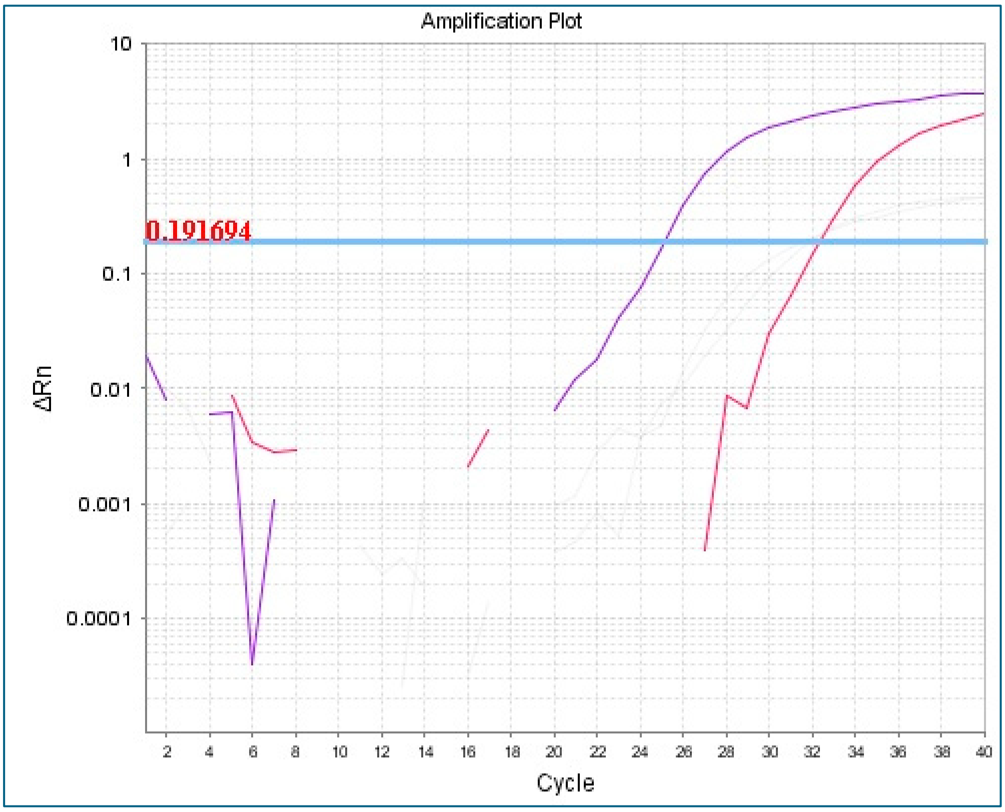

Figure S4. PCR amplification plot for *lppS* gene DNA of *Mycoplasma conjunctivae* extracted from ocular swabs of Subject 1 (fluorescence signal, ΔRn, plotted versus cycle number from a sample [purple curve] and a positive control [red curve]).

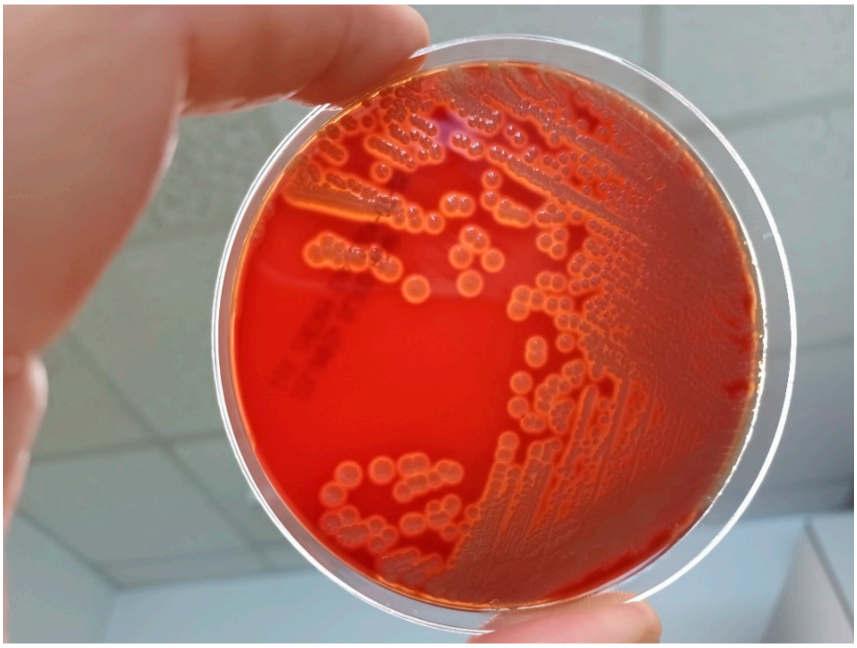

Figure S5. The hemolytic strain of EPEC *eae* positive cultured on Columbia Agar 5% Sheep Blood from the intestine of Subject 4.
